# Case Report: Virtual Reality Analgesia in an Opioid Sparing Orthopedic Outpatient Clinic Setting: A Case Study

**DOI:** 10.3389/frvir.2020.553492

**Published:** 2020-12-14

**Authors:** Reza Firoozabadi, Moamen Elhaddad, Sydney Drever, Maryam Soltani, Michael Githens, Conor P. Kleweno, Sam R. Sharar, David R. Patterson, Hunter G. Hoffman

**Affiliations:** 1 Orthopedic Trauma Surgery Clinic, Harborview Medical Center, University of Washington, Seattle, WA, United States; 2 Department of Rehabilitation Medicine, University of Washington, Seattle, WA, United States; 3 Department of Anesthesiology & Pain Medicine, School of Medicine, University of Washington, Seattle, WA, United States; 4 Department of Radiology, University of Washington, Seattle, WA, United States; 5 Department of Mechanical Engineering, College of Engineering, University of Washington, Seattle, WA, United States; 6 Department of Psychology, University of Washington, Washington, ME, United States

**Keywords:** virtual reality, pain, analgesia, ex-fix pin removal, distraction, outpatient

## Abstract

Immersive virtual reality is proving effective as a non-pharmacologic analgesic for a growing number of painful medical procedures. External fixator surgical pins provide adjunctive stability to a broken pelvic bone until the bones heal back together, then pins are removed. The purpose of the present case study was to measure for the first time, whether immersive virtual reality could be used to help reduce pain and anxiety during the orthopedic process of removing external fixator pins from a conscious patient in the orthopedic outpatient clinic, and whether it is feasible to use VR in this context. Using a within-subject within wound care design with treatment order randomized, the patient had his first ex-fix pin unscrewed and removed from his healing pelvic bone while he wore a VR helmet and explored an immersive snowy 3D computer generated world, adjunctive VR. He then had his second pin removed during no VR, standard of care pain medications. The patient reported having 43% less pain intensity, 67% less time spent thinking about pain, and 43% lower anxiety during VR vs. during No VR. In addition, the patient reported that his satisfaction with pain management was improved with the use of VR. Conducting simple orthopedic procedures using oral pain pills in an outpatient setting instead of anesthesia in the operating room greatly reduces the amount of opioids used, lowers medical costs and reduces rare but real risks of expensive complications from anesthesia including oversedation, death, and post-surgical dementia. These preliminary results suggest that immersive VR merits more attention as a potentially viable adjunctive non-pharmacologic form of treatment for acute pain and anxiety during medical procedures in the orthopedic outpatient clinic. Recent multi-billion dollar investments into R and D and mass production have made inexpensive immersive virtual reality products commercially available and cost effective for medical applications. We speculate that in the future, patients may be more willing to have minor surgery procedures in the outpatient clinic, with much lower opioid doses, while fully awake, if offered adjunctive virtual reality as a non-pharmacologic analgesic during the procedure. Additional research and development is recommended.

## INTRODUCTION

High energy pelvic fractures cause significant damage and bleeding. Mortality (death) from pelvic fractures ranges from 7.6 to 19% of the patients (Sathy et al., 2009). Orthopedic surgeons use external fixator surgical pins to provide adjunctive stability to the pelvic bone until the bones heal back together (McDonald et al., 2017). After sufficient healing has occurred (e.g., after 6 weeks) the external-fixator pins need to be removed, by having the surgeon manually unscrew the pins out of the pelvic bone using a “ratchet-wrench like” medical device, a procedure known as ex-fix pin removal. Many patients have their pins removed in the operating room, during monitored anesthesia care involving powerful sedative-hypnotics and narcotics, e.g., propofol, fentanyl, delivered intravenously by an anesthesiologist (Fragomen et al., 2018). Alternatively, this external fixator pin removal procedure can also be conducted in the outpatient clinic, without anesthesia, using standard oral pain pill medications (analgesia), greatly reducing rare but serious medical risks of anesthesia including oversedation, death, and post-surgical dementia. Pin removal in the outpatient clinic lowers medical costs by $600–$1,000 per patient because there is no operating room rental, no anesthesiologist needs to be present to continuously monitor the patient, and there are no expensive anesthesia-related post-surgical complications. Having threaded surgical pins unscrewed from their pelvic bones in an outpatient clinic while fully conscious may sound too painful or anxiety provoking for many patients. Patients carefully avoid touching the pins during the 6 weeks of bone mending. Accidently bumping the pins during the day, or during sleep, can be painful, and may delay bone fusion. So the patients are often nervous that the pin removal procedure is going to be painful and unpleasant, and many patients opt to get their pins removed in the operating room, using powerful anesthesia.

Pain intensity can be altered through psychological mechanisms (Melzack and Wall, 1965). For example, pain may be enhanced by expectations of pain or expectations of harm (Fields, 2018), attentional focus (Ploner et al., 2011), anxiety (Heathcote et al., 2017; Hemington et al., 2017) and prior learning (Noel et al., 2015a,b). Conversely, pain may be reduced by increasing patients’ expectations of analgesia (Benedetti et al., 1999; Fields, 2018), using distraction (Rischer et al., 2020), hypnotic suggestion (Patterson et al., 2006; Patterson, 2010) or relaxation (Syrjala et al., 1995).

The multidimensional nature of pain results in possibilities to reduce it through, for example, manipulation of anxiety or attentional processes. One especially promising psychological treatment, adjunctive immersive virtual reality distraction, has been shown to reduce pain during painful medical procedures (Hoffman, 1998, 2004; Hoffman et al., 2000, 2019a,b; Gold et al., 2006; Sharar et al., 2007; Li et al., 2011; Garrett et al., 2014; Atzori et al., 2018a,b; Spiegel et al., 2019; Al-Ghamdi et al., 2020). The logic for how VR reduces pain is as follows. Patients look into a head mounted display, and their attention is directed into the computer generated world. Humans are visually dominant. VR technology makes the patient’s brain integrate multisensory (visual, auditory and sometimes tactile and olfactory) computer generated sensory distractions. VR diverts the patient’s attention away from the painful procedure, thus leaving less attention available for pain perception (Hoffman, 1998; Hoffman et al., 2000, 2004, 2007; Gold et al., 2006, 2007; Garrett et al., 2014; Birnie et al., 2017).

Adjunctive immersive Virtual Reality (VR) has been shown to be an effective means of reducing pain during medical procedures in a number of non-orthopedic settings (e.g., severe pediatric burn wound care in the ICU, (Hoffman et al., 2019b). The purpose of the present novel case study was to measure for the first time, whether immersive virtual reality could be used to help reduce pain and anxiety during the minor surgical process of removing external fixator pins from the pelvic bone of a conscious patient in the orthopedic outpatient clinic, and whether it is feasible to use VR in this context. If, in the future, VR increases patients’ likelihood of deciding to have their pins removed in the outpatient clinic instead of the OR, this will result in substantial opioid sparing and cost savings.

## MATERIALS AND METHODS

### Subjects

This research was conducted in accordance with the Declaration of the World Medical Association (www.wma.net). The subject gave written informed consent in accordance with the Declaration of Helsinki. Both written and verbal informed consent were obtained using a protocol approved by the University of Washington Human Subjects Review Committee. STUDY00002673, Ethics Committee Team D.

While repairing his roof, the athletic Caucasian male patient in his mid 50s, slipped and fell three stories onto his concrete driveway. He landed feet first, sustaining traumatic pelvic fractures, and hemodynamic instability, potentially life-threatening injuries. He also broke his forearm and wrist, and had bilateral calcaneous fractures. The patient was airlifted to a regional trauma center via medical helicopter, where he was put into a medically induced coma for several days, and received seven surgeries before regaining consciousness. For his pelvic ring bone injury, he underwent percutaneous management of his posterior lower pelvic ring and placement of an external fixator across the anterior upper aspect of his pelvis. The external fixator consisted of two 5 millimeter pins placed roughly 15 cm deep into his pelvis through the anterior inferior iliac spine, directed cranial to the sciatic notch. These external fixator pins were connected with clamps and a bar exterior to his skin. The pins protruded several inches out of his body at waist level, and the medical pins were held in place outside of his body by a precision medical bar clamp system. The purpose of the external fixator was to hold the two pieces of broken pelvic bone back together, and to provide stability to his anterior pelvis while healing occurred, typically for ~6 weeks. At the time of the current study, the pelvis had sufficiently healed back together, and as a result it was time to remove the pins.

Although many patients opt to have the procedure conducted in the operating room under anesthesia (i.e., while unconscious), the current patient opted to undergo routine removal of two pelvic external fixator pins without anesthesia, in the outpatient clinic of a regional trauma center. Following standard of care, the patient took a 5 mg Oxycodone pain pill orally, prior to the outpatient procedure. Because of the use of the within subject, within-wound care design, the amount of pain medications in the patient’s bloodstream was essentially identical during the two pin removals.

The two ex-fix pins were removed during a single visit. Neither pin appeared infected or loose, and the surgeon noted that the two pins were similar in consistency, i.e., similarly difficult to unscrew. Using a within-subject design with treatment order randomized, the patient had his first pin removed from his pelvic bone during VR, i.e., standard of care pain pill medications plus VR, and he had the second pin removed during no VR, i.e., standard of care pain pill medications. Using graphic rating scales, after both pins had been removed, the patient rated his pain, anxiety and satisfaction with his pain management during pin removal comparing No VR vs. VR.

During virtual reality, the patient wore a pair of head mounted virtual reality goggles, and interacted with virtual objects in virtual reality using a mouse. The VR software computer program and VR hardware were designed to give the patient the illusion of going into the computer generated world as if it was a place he was visiting (Slater et al., 1994).

The VR system was comprised of a gaming laptop: MSI GeForce GTX 1080 8 GB, Intel Core i7 7th (2.80 GHz), 16 GB RAM, Windows 10 operating system connected to an HTC VIVE VR helmet with FOV 110 degrees, with 1,080 × 1,200 pixels per eye resolution and a refresh rate of 90 Hz. The patient wore Bose Q35 noise canceling earphones plugged into the gamers laptop, to make the system more immersive.

The HTC Vive VR system required Steam VR “seated user” setup and calibration of two HTC VIVE position tracking base stations before the patient’s arrival. Each base station was mounted onto a portable tripod, which held each base station at a height of 6 feet. The base stations were placed in two corners of the hospital clinic room, facing the patient, and the tracking base station power adapters were plugged into electrical outlets. The patient was positioned such that at least one of the base stations would be able to “see” the VR helmet at all times. USB and HDMI cables from the VR helmet were plugged into the portable gamers laptop computer. Setting up and starting up the portable VR system took ~20 min before the patient arrived. A researcher on our team did all of the VR system setup. Once the VR system was set up, the patient was shown by the researcher, how to left click the wireless mouse to throw snowballs, the patient put on the VR helmet and was told to “throw a lot of snowballs at any virtual objects he wanted.” The patient was allowed to play VR for 2 min before the pin removal began.

**FIGURE 1 |:**
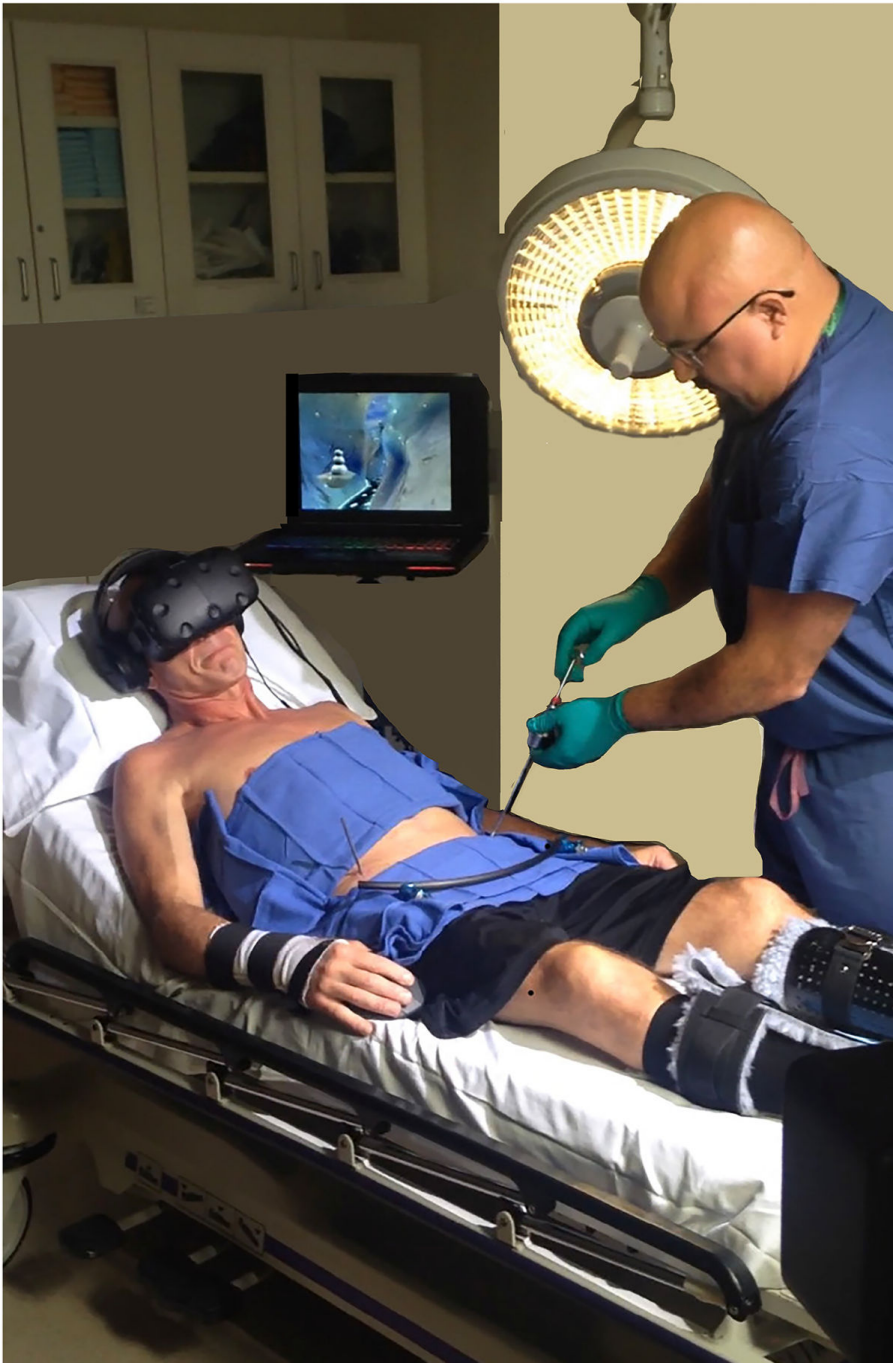
A patient in VR during external fixator removal. Photo and copyright Hunter Hoffman, www.vrpain.com.

During the VR condition, the patient wore a HTC VIVE VR head-mounted display. In VR, the patient could see a virtual arctic canyon, complete with flowing river below, blue sky above, and terraced canyon walls to the sides of the canyon containing virtual penguins, igloos, and snowmen. During VR, the patient “glided” through the virtual world along a predetermined path, and he could target and shoot virtual objects in the virtual world using his head tracked VR head orientation and/or mouse tracking to aim and left click the mouse button to shoot snowballs. The patient could hear sound effects as they interacted with objects in VR via the snowballs, mixed with music from Paul Simon’s “Graceland” playing in the background. The snowy virtual environment was originally designed to treat severe burn patients during painful wound care sessions requiring the patients to remain motionless during the procedure. The custom VR software allows researchers to quickly adjust the VR horizon of the VR world (by hitting the up or down arrow on the keyboard) so the patient could see the river and float through the 3D valley, even if the patient was in a semi reclined posture, typical for ex-fix removal, see Figure 1. The VR analgesia software is custom designed to be easy for patients to use with minimal instructions and minimal practice, and does not require previous video gaming experience. The walls of the canyon encourage patients to minimize excessive head movements. The snow is designed to be associated with positive memories and emotions, while being computationally inexpensive, to allow fast frame rate to help minimize simulator side effects. The Paul Simon Graceland background music is upbeat and was added at Paul Simon’s suggestion, to help put the patient in a positive mood, mixed in with sound effects from interactions with the VR world.

A disposable HTC VIVE foam face pad was used and discarded after use. The equipment was systematically disinfected using chemical disinfectants. As a precaution, to help maximize sterility, the surgeon never touched the VR equipment. A medical resident was dedicated to managing the VR analgesia equipment and collecting the pain ratings.

The patient had two ex-fix pins removed, one after the other, with a 5 min “wash out” period between pins. Treatment order was randomly assigned, using the random.org website. The patient was randomized to receive Yes VR, standard of care + VR, during his first pin removal, and “No VR,” standard of care, during the second pin removal. To minimize interference with the surgical procedure, and to allow the usual sequence of events of a typical surgery procedure, the patient was not asked any questions during the procedure. After both pins had been removed, the patient received the following instructions prior to answering the set of five separate questions. “Please indicate how you felt during pin removal today by making a mark anywhere on the line. Your response doesn’t have to be a whole number.”

After the pin removal session, the patient rated his pain using Graphic Rating Scales (GRS). The GRS tool was used to assess three reports of the pain experience, “worst pain,” “pain unpleasantness,” and “time spent thinking about pain” that correspond to three separable components of the pain experience; sensory pain, affective pain, and cognitive pain, respectively. The GRS is a 10-unit horizontal line labeled with number and word descriptors. Descriptor labels were associated with each mark to help the respondent rate pain magnitude in each domain. For worst pain, the GRS descriptors were *no pain at all, mild pain, moderate pain, severe pain,* and *excruciating pain*. For pain unpleasantness, the GRS descriptors were *not unpleasant at all, mildly unpleasant, moderately unpleasant, severely unpleasant,* and *excruciatingly unpleasant.* For time spent thinking about pain, the GRS descriptors were *none of the time, some of the time, half of the time, most of the time, all of the time.* For nervous, the GRS descriptors were *no anxiety at all, mild anxiety, moderate anxiety, severe anxiety, excruciating anxiety*. Such pain rating scales have been shown to be valid through their strong associations with other measures of pain intensity, as well as through their ability to detect treatment effects (Jensen and Karoly, 2001; Jensen, 2003; Hoffman et al., 2004). The patient also rated how satisfied he was with his pain management during No VR vs. during VR, with descriptors *completely unsatisfied, mostly unsatisfied, half satisfied, mostly satisfied, completely satisfied*.

### Experimental Design

For the current case study, a within-subject, within-wound care design was used (Maani et al., 2011; Khadra et al., 2018, 2020; Hoffman et al., 2019b). During VR, the patient interacted with a 3D snowy canyon in virtual reality during his first pin removal, vs. No VR during his second pin removal, treatment order randomized using a number sequence from random.org. The primary outcome measure was the patient’s worst pain during VR (usual standard of care pain medications + VR) during the first ex-fix pin removal, vs. their worst pain during No VR (usual standard of care pain medications) during the second pin removal during the same outpatient clinic session.

During VR, the patient went into a 3D computer generated snowy VR environment, where he interacted with snowmen, igloos, penguins, and other virtual objects by throwing snowballs by left clicking a wireless computer mouse. After the two pins were removed, the patient briefly rated how much pain he had experienced during No VR vs. during Yes VR using graphic rating scales.

## RESULTS

**FIGURE 2 |:**
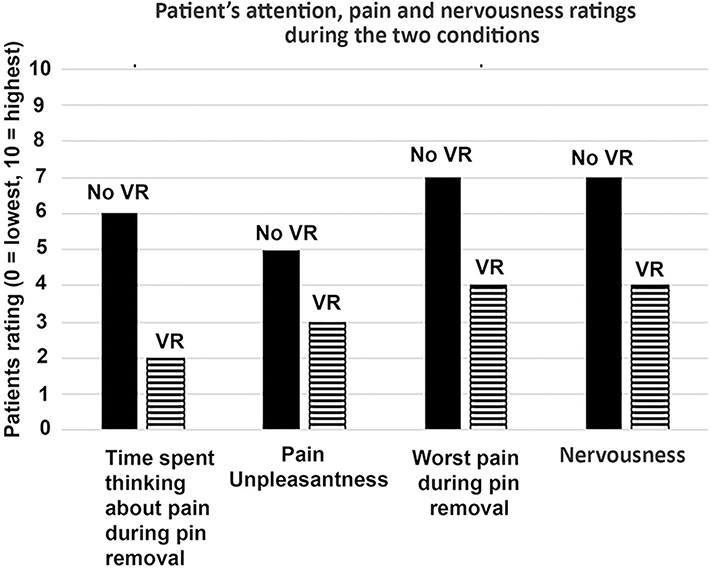
Patient rated their pain during one pin removal with VR, vs. another pin removal during No VR.

The patients’ ratings relating to attention, pain and nervousness given by the patient during the two conditions are shown in Figure 2.

The patient reported having 43% less pain intensity, spent 67% less time thinking about his pain, and reported 43% lower anxiety during VR vs. during No VR. In addition, the patient reported that his satisfaction with pain management was improved with the use of VR. He was “satisfied” with his standard of care pain management during No VR. And this increased to “very satisfied” during pin removal during virtual reality. The patient said he would get his pins removed in the clinic again next time, whether VR was available or not. But he would prefer to receive VR, and he would be willing to pay “a few hundred dollars” for VR next time. He also said the availability of VR made him “absolutely” more likely to recommend the University of Washington Medical Center to a friend. The surgeon performing the ex-fix pin removals, author RF, noted that the two pins had the same consistency, and were approximately equally difficult to remove. Although the entire visit (including pre-surgery X-rays) took ~1 h, the actual amount of time it took to unscrew the first pin was 1.2 min for VR. After the first pin was removed during VR, the surgeon applied a sterilized gauss bandage to the wound until with excision wound stopped bleeding. The washout period between completing removal of the first pin and beginning removal of the second pin was 5 min. The surgeon walked around to the other side of the patient, and had his surgical instruments brought over to that side of the patient. The surgeon removed the second pin with no VR. The actual amount of time unscrewing the No VR pin was 1.6 min. During discussion with the patient after the study was completed, the patient remarked that during VR, he was surprised when the doctor said the VR pin removal was done. He said “I was expecting a lot more pain.” He said that “during VR, I was focusing entirely on throwing snowballs at snowmen and penguins, a pleasant distraction.” He said that “during the second pin with No VR, I did not have that distraction, and my mind wandered onto the pain itself. That got pretty intense.”

## GENERAL DISCUSSION

Ex-fix pin removal is a surgical procedure routinely conducted in the operating room, using monitored anesthesia care involving powerful sedative-hypnotics and narcotics, e.g., propofol, fentanyl, delivered intravenously by an anesthesiologist. Getting their simple orthopedic procedures in an outpatient setting instead of the operating room greatly reduces the amount of opioids used (opioid sparing), lowers medical costs and reduces rare but real risks of expensive complications from anesthesia including oversedation, death, and post-surgical dementia. The drawback is that pin removal in the outpatient clinic with only oral pain pills for pain medication is often painful, and anxiety provoking.

The current study is the first published study to explore the feasibility of using virtual reality as an adjunctive non-pharmacologic analgesic during ex-fix pin removal in the outpatient clinic (non-operating room). The patient reported having 43% less pain intensity and 43% lower anxiety during ex-fix pin removal during VR vs. ex-fix pin removal during No VR. In addition, the patient reported that his satisfaction with pain management was improved with the use of VR.

Although the mechanism of VR analgesia remains an important research topic, theories typically center around distraction (Hoffman et al., 2000; Birnie et al., 2017). These theories posit that humans have limited conscious attentional resources (Kahneman, 1973; Schneider and Shiffrin, 1977; Shiffrin and Schneider, 1977) such that when two activities demand more conscious attention capacity than is available, “concurrent activities are likely to be mutually interfering” (Kahneman, 1973, p. 11). Pain requires conscious attention (Eccleston, 1994; Chapman and Nakamura, 1998; Eccleston and Crombez, 1999), and according to McCaul and Malott (1984), the subjective experience of pain from the painful stimulus requires paying attention to a painful stimulus. If patients focus their attention on non-pain stimuli (such as VR), while in virtual reality, less attentional resources are available to process the sensory input from the pain stimulus, reducing the subjective experience of pain intensity (see McCaul and Malott, 1984; Eccleston and Crombez, 1999). Virtual reality non-pharmacologically reduces the attentional resources available to process incoming signals from a pain stimulus.

### LIMITATIONS

Several limitations of the current study should be taken into consideration when interpreting the results. Neither the surgeon nor the patient were blinded to the treatment conditions. This study was a self-controlled comparison of treatment condition vs. control condition, in which the patient served as his own control (Louis et al., 1984). One issue of sequential within-subject design studies is that one treatment may influence or contaminate the following condition (e.g., the control condition in this case). The current study used a 5 min “wash out” period between pin removals, to help reduce carry over effects. In the current study, the patient was randomized to receive VR for his first pin removal and No VR for his second pin removal. If there was a carry over effect (i.e., if VR continued to reduce pain during the second “no VR” treatment condition), that would likely lead to a conservative underestimation of how much VR reduced pain in the current case study. Case studies are inherently scientifically inconclusive (Campbell and Stanley, 1963). Lack of generalizability is a known limitation of case studies. It is not possible to know (from a case study) whether the current results showing reduced pain during VR will generalize to other patients who receive ex-fix pin removals. Furthermore, since this patient had never used VR before, the novelty may have made VR more effective. However, a number of previous studies have shown that VR continues to be effective when used during several painful medical procedures per patient, on different study days (e.g., Hoffman et al., 2019a). Large randomized controlled studies will be needed to determine whether VR analgesia is effective during ex-fix pin removal. Virtual Reality analgesia also has potential applications for a wide range of painful and anxiety provoking orthopedic medical procedures and rehabilitation (Steele et al., 2003) e.g., using a circular saw to remove a child’s cast from a healed broken arm. Spiegel et al. (2019) report use of VR analgesia during a wide range of medical procedures including unspecified orthopedic procedures.

The current study compared adjunctive VR to the standard of care condition. Full distraction vs. little or no distraction addresses the practical question of whether patients benefit from VR compared to what they are currently receiving during pin removal in the outpatient clinic. Future studies should consider comparing VR with other types of controlled distractions, e.g., a less immersive version of the same VR world (e.g., Al-Ghamdi et al., 2020), or comparing immersive VR to watching TV (Spiegel et al., 2019), or a study comparing immersive VR to a PC tablet (Le May et al., 2020, proposed/in progress). Ideally the patients in the high tech VR group would not know there is a low tech VR group, and patients in the low tech VR group would not know there is a high tech VR group, and it is ideal if the researcher (or at least the research assistant interacting with the patient) can remain blind to treatment group, a double-blind design, however such rigorous designs in the clinical setting are challenging, and require large sample sizes.

For the current patient, according to the orthopedic surgeon who removed the pins (author R.F.), the two pins were approximately average difficulty to unscrew, and had similar consistency. In contrast, in our experience, pin removals are sometimes more difficult to unscrew. Sometimes the pins get somewhat stuck in the bone, and require extra torque from the hand held medical rachet wrench used by the surgeon to break the pin loose from the bone it is screwed into. And it is often not possible to predict in advance how difficult a pin is going to be to remove. In the current case study, the patient tolerated pin removal well, and the VR system was sufficiently distracting. His worst pain rating during No VR was severe (but not excruciating), and his worst pain rating during VR was mild. The current VR system worked well for this patient. However, for some patients, a much stronger, much more distracting version of virtual reality may be needed. Virtual reality and pain are in a divided attention tug of war over the patient’s limited attentional resources. If the medical procedure sends more nociceptive signals to the brain (e.g., if the pin gets stuck in the bone), a more immersive virtual reality experience may be needed to keep the patient’s attention focused on the VR.

**FIGURE 3 |:**
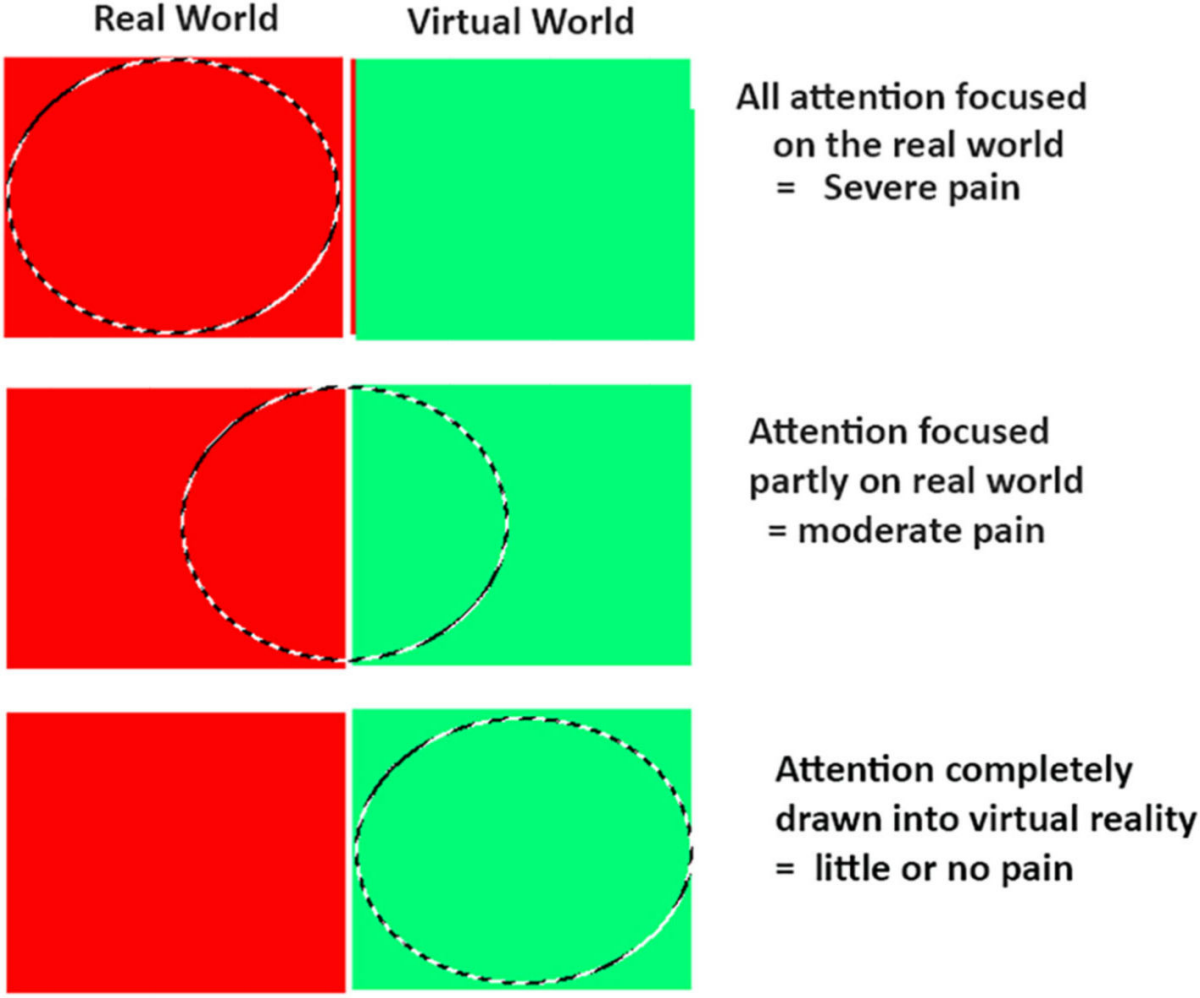
Hoffman et al. (2000) proposed that the patient’s illusion of “being there” in VR, leaves less attention available to process incoming nociceptive signals. Although not conclusive, the current patient’s pattern of results are consistent with an attentional mechanism. Copyright Hunter Hoffman, www.vrpain.com.

In designing the first immersive VR analgesia system, Hoffman et al. (2000) predicted that the illusion of “being there” in the 3D computer generated environment, interacting with virtual objects, and getting converging multisensory evidence consistent with the notion that they are “there” in VR, would be unusually attention demanding, and thus unusually effective for pain distraction (see diagram in Figure 3). Although the current patient showed large reductions in pain and anxiety during VR, more research and development is needed to further increase the “dose” of virtual reality distraction available. VR systems designed to increase the patients illusion of “being there” in virtual reality, have been shown to increase how effectively VR reduces pain (Hoffman et al., 2006; Dahlquist et al., 2007; Wender et al., 2009; Al-Ghamdi et al., 2020). Increasing the immersiveness of the VR system has been shown to increase the amount that VR reduces pain. For example, in laboratory studies, a VR helmet with a wider field of view, that stimulates more peripheral vision, was significantly more effective at reducing pain than a narrow field of view VR helmet (Hoffman et al., 2006). It is possible that an extra wide field of view VR helmet such as the VRGineering XTAL helmet with 180 degrees field of view would be more effective at reducing pain than the 110 degree field of view VR helmet used in the current study, and the XTAL has its own helmet-mounted inertial head tracking system and thus does not require extra motion tracking base stations. Other previous laboratory studies have shown that interactive VR was more effective at reducing pain than passive VR (Dahlquist et al., 2007; Wender et al., 2009; Al-Ghamdi et al., 2020). Although the current VR system involved interactivity, it is likely that future systems with much more interactivity (e.g., with eyetracking, Al-Ghamdi et al., 2020 and tactile feedback, Hoffman, 1998, 2004; Hoffman et al., 1998) will be more distracting and more effective at reducing pain than the current standard VR system. Custom designed VR worlds specifically designed for VR analgesia are ideal, because it is important for the patient to interact with the objects in VR while keeping their torso still during the ex-fix pin removal. And it is important for patients to be able to sit on a partially reclined bed during the procedure.

The VR system used in the current study has some practical limitations worth considering. The current VR system hardware consisted of a fast gamers laptop with a specialized video card designed to be used for virtual reality. The VR goggles plugged into the laptop. Two helmet tracking HTC VIVE base stations had to be temporarily mounted on tripods and plugged into electrical outlets, in the outpatient treatment room. Our VR system required calibration of the VR tracking base station cubes prior to the patient’s arrival, and the VR software had to be adjusted slightly depending on the amount of inclination of the patient. The VR system used in the current study was a research system designed to test the feasibility of whether a patient could even use VR during an orthopedic pin removal procedure.

Although the Oculus Quest 2 was not used in the current study, the Oculus Quest 2 VR helmet does not require any tracking base stations, the stand-alone helmet does not require any laptop, and does not even have any wires. The Oculus Quest 2 is completely wireless, untethered and inexpensive. The VR software is downloaded into memory storage in the VR goggles themselves. For many painful medical procedures (i.e., for patients able to wear the VR helmet), this new generation of wireless VR helmets will dramatically increase how easy and inexpensive it is to use VR analgesia. However, the spike in pain during ex-fix pin removal is often brief but intense, so although a wireless VR helmet (e.g., Oculus Quest 2) is much cheaper and simpler, a more immersive, extra wide field of view, extra high resolution, medical strength VR helmet such as the VRgineering XTAL VR helmet could *in theory* increase the chances that the VR system will be distracting enough to hold the patient’s attention even during more difficult pin removal sessions, and to help the patient have the most positive experience possible, an important consideration.

There is a growing literature of research studies exploring the use of virtual reality analgesia during painful medical procedures (acute pain). The present study is novel in that it is one of the first studies to expand VR analgesia into orthopedic patient populations (see also Steele et al., 2003; Spiegel et al., 2019, Le May et al., 2020, in progress). As far as the authors are aware, the current original study is the first published study to focus on the feasibility of using VR during ex-fix pin removal. The finding that VR is feasible in this orthopedic setting helps open the door to more widespread use of VR during orthopedic medical procedures. If VR increases patients’ likelihood of deciding to have their pins removed in the outpatient clinic instead of the OR, this will result in substantial opioid sparing and cost-savings.

## FUTURE DIRECTIONS

Conducting simple orthopedic procedures in an outpatient setting greatly reduces the amount of opioids used, lowers medical costs and reduces rare but real risks of expensive complications from anesthesia including oversedation, death, and post-surgical dementia. These preliminary results suggest that immersive VR merits more attention as a potentially viable adjunctive non-pharmacologic form of treatment for acute pain and anxiety during medical procedures in the orthopedic outpatient clinic. Recent multi-billion dollar investments into R&D and mass production have made inexpensive immersive virtual reality products commercially available and cost effective for medical applications. VR has recently become more immersive at a much more affordable price (e.g., $35,600 per 90 degree FOV helmet in 2013 vs. $299 per helmet for a 110 degree FOV helmet in 2020), increasing potential for dissemination. We speculate that in the future, patients may be more willing to have minor surgery procedures in the outpatient clinic, with much lower opioid doses, while fully awake, if offered adjunctive virtual reality as a non-pharmacologic analgesic during the procedure. With growing concerns about the epidemic level of opioid overdose deaths in the United States (Chen et al., 2019), research and development of adjunctive non-opioid pain management techniques has become a national priority (Keefe et al., 2018). Virtual Reality analgesia appears to be an especially promising non-opioid technique (Keefe et al., 2012), Additional research and development is recommended.

## APPENDIX

Appendix 1a. Here is the timeline of a typical patient getting their pins removed with anesthesia in the Operation Room.

- Arrive to the hospital,
- The patient puts on a gown and is checked into the preoperative area.
- Patient lies down on a gurney.
- Patient has X-rays taken of their pelvic bone.
- The surgeon double checks the x-rays to make sure the bone has healed sufficiently to remove the ex-fix pins.
- Medical staff takes the patient into the OR
- Anethesiologist inserts an IV, anesthestizes the patient
- Once the patient is anesthetized, the surgeon removes the pins, while the anesthesiologist monitors vital signs and adjusts the anesthesia dose.
- The Anesthesiologist brings the patient back to a state of full alertness.
- Gourney takes patient to postoperative are.
- Patients recovers in the postoperative area for 1–2 h and is discharged home.

Appendix 1b. Here is the timeline of a typical patient getting their pins removed with analgesia in the outpatient clinic instead of the Operating Room.

- For pin removal in the outpatient clinic.
- Patient arrives.
- Patient lies down on a gurney.
- Patient has X-rays taken of their pelvic bone.
- The surgeon double checks the x-rays to make sure the bone has healed sufficiently to remove the ex-fix pins.
- The patient is brought into the outpatient clinic room
- No operating room is needed, and no anesthesiologist is required.
- The doctor removes the pins.
- Patients recovers in the postoperative area for 30 min to an hour and is discharged home.
